# BiZact versus cold steel for post-tonsillectomy hemorrhage: a multicenter randomized trial

**DOI:** 10.1007/s00405-025-09703-3

**Published:** 2025-10-13

**Authors:** Martin Mølhave, Nina M. Lyhne, Dennis F. Jensen, Susanne H. Nielsen, Pernille Hahn, Jesper K. Holm, Therese Ovesen, Jannik Bertelsen

**Affiliations:** 1 Department of Otorhinolaryngology, Head & Neck Surgery, University Clinic for Flavour, Balance and Sleep, Gødstrup Hospital, Hospitalsparken 15, Gødstrup, Herning, Central Denmark Region 7400 Denmark; 2 Aarhus University, Aarhus, Denmark; 3 Department of Oto-Rhino-Laryngology, Head and Neck Surgery, Aalborg University Hospital, Aalborg and Thisted, Denmark; 4 Department of Oto-Rhino-Laryngology, Head and Neck Surgery, Regional Hospital of Southern Jutland, Sønderborg, Denmark; 5 Department of Oto-Rhino-Laryngology, Head and Neck Surgery, Lillebaelt Regional Hospital, Vejle, Denmark; 6 Department of Oto-Rhino-Laryngology, Head and Neck Surgery, University Hospital of Southern Denmark, Esbjerg, Denmark; 7 Department of Clinical Medicine, Aarhus University, Aarhus, Denmark

**Keywords:** Tonsillectomy, Postoperative hemorrhage, Randomized controlled trial, Surgical complications

## Abstract

**Purpose:**

To compare post-tonsillectomy hemorrhage (PTH) rates and severity between BiZact and cold steel tonsillectomy in a multicenter randomized controlled trial (RCT).

**Methods:**

This non-inferiority RCT was conducted at five Danish hospitals between June 2022 and May 2024. Patients aged ≥ 4 years undergoing tonsillectomy for benign indications were randomized to BiZact or cold steel dissection. Patients and caregivers were blinded to group assignment. Primary outcome was PTH within 30 days, categorized by severity: spontaneous hemostasis, treatment in local anesthesia, or general anesthesia. Secondary outcomes included operative time, blood loss, infection, and readmission due to pain. Modified Poisson regression with robust standard errors was used to estimate relative risk (RR) in an intention-to-treat analysis.

**Results:**

Of 2,750 screened patients, 1,250 were randomized: 645 to BiZact and 605 to cold steel. Among 1,187 patients analyzed, return to theatre occurred in 5% of BiZact patients and 10% of cold steel patients (*p* = 0.004). Bleeding managed in local anesthesia occurred in 7% in both groups; spontaneous hemostasis occurred in 3% versus 5%, respectively. Overall PTH rates were 12% for BiZact and 17% for cold steel (*p* = 0.02). Adjusted RR for any PTH was 0.70 (95% CI: 0.53–0.92). No complications beyond predefined outcomes occurred.

**Conclusion:**

BiZact tonsillectomy was associated with a lower risk of severe and overall PTH compared to cold steel. Comprehensive bleeding documentation may account for the higher absolute PTH rates compared to previous studies.

**Trial registration:**

Registered at ClinicalTrials.gov, ID NCT05270109. Registered March 7, 2022.

**Supplementary Information:**

The online version contains supplementary material available at 10.1007/s00405-025-09703-3.

## Introduction

Tonsillectomy is one of the most frequently performed ENT surgeries worldwide, with over 8,000 procedures conducted annually in Denmark alone [1]. The primary indications include recurrent acute tonsillitis and upper airway obstruction [2]. Despite its routine nature, tonsillectomy is associated with considerable morbidity, most notably post-tonsillectomy hemorrhage (PTH) and postoperative pain [2, 3]. 

PTH is typically classified as either primary (occurring within 24 h postoperatively) or secondary (occurring after 24 h) [3–6]. A recent meta-analysis reported an overall PTH incidence of 4.6% (95% CI: 2.1–8.0) including 9% for cold steel tonsillectomy [7], although substantial heterogeneity exists across studies due to differences in surgical techniques, definitions of PTH, and study design [4–6, 8]. Self-limiting bleeding events (such as blood-tinged sputum) often remain unrecorded, resulting in potential underreporting [4–7, 9]. In contrast, severe PTH may necessitate operative intervention and can be life-threatening [6, 10]. Additionally, postoperative pain is common and may lead to prolonged hospital stay or readmission for pain control and hydration [10]. 

A range of instruments are used for tonsillar dissection and hemostasis, broadly categorized as “cold” techniques (without thermal energy) and “hot” techniques (involving thermal coagulation). Evidence suggests that instrument choice influences PTH risk. Cold steel tonsillectomy has been associated with lower bleeding rates compared to hot techniques [3–6, 10–14], and is considered the standard approach in Denmark [3, 6]. 

The BiZact device is a single-use, bipolar, impedance-sensitive tissue sealer designed to deliver targeted energy to tissue grasped between its jaws. By limiting lateral thermal spread, the device aims to reduce collateral damage during tonsillar dissection [15, 16]. Early clinical studies have reported low PTH rates following BiZact tonsillectomy [17]. However, a recent meta-analysis found no significant reduction in bleeding rates compared to other methods [16], and prior Randomized controlled trials (RCTs) have been limited by small sample sizes [18, 19]. Therefore, the BiZact device was selected for comparison with cold steel because a large-scale RCT on the device was lacking in the literature [17]. 

Given the limited high-quality evidence on BiZact, especially in comparison with cold steel dissection, this study aimed to compare PTH outcomes between BiZact and cold steel tonsillectomy in a large, multicenter randomized controlled trial. We hypothesized that BiZact would be non-inferior to cold steel with respect to PTH risk.

## Methods

### Study design and data sources

This was a multicenter, parallel-group, patient-blinded, RCT comparing the BiZact Tonsillectomy Device with cold steel tonsillectomy in terms of PTH. The study protocol has been published [20]. Data were collected from the Danish Tonsil Database, a national quality register. The study was conducted in accordance with the CONSORT 2010 guidelines [21, 22], with a completed checklist available (Table S1). The study was registered on ClinicalTrials.gov (NCT05270109) before patient inclusion began. A prespecified interim analysis was conducted at 50% inclusion. No safety concerns were identified, and no changes were made to the protocol or allocation strategy.

### Power considerations

The required sample size was estimated to be 1,250 patients, based on an anticipated PTH rate derived from a 2018 preliminary study of Danish tonsillectomy patients at a secondary center predominantly utilizing the cold steel technique. This study, involving 243 patients, reported a 7.9% overall PTH rate necessitating general anesthesia [23]. Data was registered in the Danish Tonsil Database. The sample size calculation used a non-inferiority margin of 4%, a significance level of 0.05, a statistical power of 80%, and estimated a 10% dropout rate.

### Randomization

Randomization was performed through the Danish Tonsil Database using a computer-generated 1:1 allocation sequence, stratified by hospital (2,000 randomizations per center). Patients were allocated to BiZact or cold steel tonsillectomy. Participants and postoperative care providers were blinded to group allocation. Surgeons were informed of the instrument used on the day prior to surgery but remained blinded to trial-wide outcome trends. Authors were blinded to all outcome data until final analysis.

### Study population

The trial was conducted across five Danish otolaryngology departments: Gødstrup Hospital (Herning), Aalborg University Hospital (Aalborg and Thisted), Regional Hospital of Southern Jutland (Sønderborg), Lillebælt Hospital (Vejle), and University Hospital of Southern Denmark (Esbjerg). Patients undergoing elective or acute tonsillectomy for benign indications between June 1, 2022, and May 31, 2024, were assessed for eligibility.

Inclusion criteria were: age ≥ 4 years, body weight ≥ 16 kg, and at least one of the following indications: tonsillar hypertrophy (including obstructive sleep apnea), recurrent tonsillitis, current or prior peritonsillar abscess (PTA), chronic tonsillitis, tonsillar plugs, residual tonsil tissue, systemic complications of group A streptococcal infection, or PFAPA syndrome. Written informed consent was required.

Exclusion criteria included known coagulopathy, anticoagulant use, concurrent soft tissue pharyngeal surgery, pharyngeal abscess other than PTA, suspected or confirmed malignancy, inability to provide informed consent, or lack of Danish language proficiency.

### Intervention

All procedures were performed using an extracapsular dissection technique. In Denmark, extracapsular tonsillectomy remains the standard procedure for infectious indications in both children and adults. For hypertrophy alone, tonsillotomy is generally preferred as first-line treatment, but patients in this trial had indications where extracapsular tonsillectomy was clinically indicated.

At sites where standard practice included adrenaline infiltration, this was administered to the palatal arch prior to dissection. In the BiZact group, tonsils were dissected along the capsule and detached at the caudal pole using the BiZact device. In the cold steel group, dissection was initiated via incision of the palatal arch followed by capsular dissection using conventional cold instruments, with hemostasis achieved by ligature, bipolar diathermy, or compression sutures as needed.

Intraoperative blood loss was quantified using a standardized gauze-weighing method. This included irrigation of the oropharynx with saline and suction through both nasal cavities prior to gag removal. Operation time was defined from Boyle-Davis gag placement to removal.

### Outcome

The primary outcome was the occurrence of PTH within 30 days, classified by severity as: Spontaneous hemostasis: Minor bleeding confirmed by ENT examination that resolved without active intervention. Treatment in local anesthesia: Events requiring bedside intervention (e.g., chemical cautery) not involving general anesthesia. Return to theatre under general anesthesia: Bleeding events requiring operative intervention and considered the most severe.

Classification was based on clinical evaluation, bleeding severity, patient cooperation, and age. All events were prospectively recorded by ENT surgeons and confirmed using standardized documentation in the Danish Tonsil Database and hospital records.

Secondary outcomes included intraoperative blood loss, operation time, postoperative infection requiring antibiotics or admission, and readmission due to pain.

### Follow-up

All outcome and exposure variables were extracted from the Danish Tonsil Database and supplemented by systematic manual review of electronic health records. To ensure data completeness and correct classification of bleeding events, all patient records were reviewed independently by the first and third authors after trial completion.

### Statistical analysis

All analyses followed an intention-to-treat approach. Categorical variables were compared using χ² or Fisher’s exact test. Continuous variables were assessed for normality and compared using Student’s t-test or Mann–Whitney U test, as appropriate.

Relative risks (RR) for PTH were estimated using Poisson regression with a log link. Robust (HC0) standard errors were applied to account for mild overdispersion (dispersion ratio = 1.49). Models were adjusted for sex, age group (4–11, 12–17, ≥ 18 years), smoking status, comorbidity, surgical indication, and treatment center. Model fit was evaluated by residual deviance. Sensitivity analyses using a quasi-Poisson model confirmed estimate robustness. Multilevel Poisson regression with a random intercept for hospital was used to test for center-level clustering (variance = 0.15). Model coefficients remained stable, supporting the use of fixed effects. Surgeon-level clustering could not be modeled due to estimation failure. All analyses were performed using RStudio. A significance level of 0.05 was applied. No adjustments were made for multiple comparisons, as only one prespecified primary outcome was tested.

### Ethical approval

The study was approved by the Danish Data Protection Agency (Case: 1–16-02–152−22) and the Regional Committee on Health Research Ethics (Case: 2202151). Data processor (case number of the main agreement 1–52-81–216−21) and cooperation agreements (reference number 1–10-81–95−21) were signed by all participating Departments. The trial adhered to the principles of the Declaration of Helsinki. Written informed consent was obtained from all participants or guardians.

The study was conducted according to Helsinki declaration. Participation was voluntary. Patients received oral and written information. Confidentiality was guaranteed. A written consent was signed by each patient.

A sponsor agreement was signed with Medtronic, the BiZact manufacturer. Support received under this agreement was for legitimate purposes only and within Danish legal guidelines. Support was provided for research purposes only, including provision of BiZact devices, generators, and scales for intraoperative measurements, with no involvement in study design, conduct, data analysis, or interpretation. No conflicting obligations exist. The research was not for commercial market approval.

## Results

### Study population characteristics

**Fig. 1:**
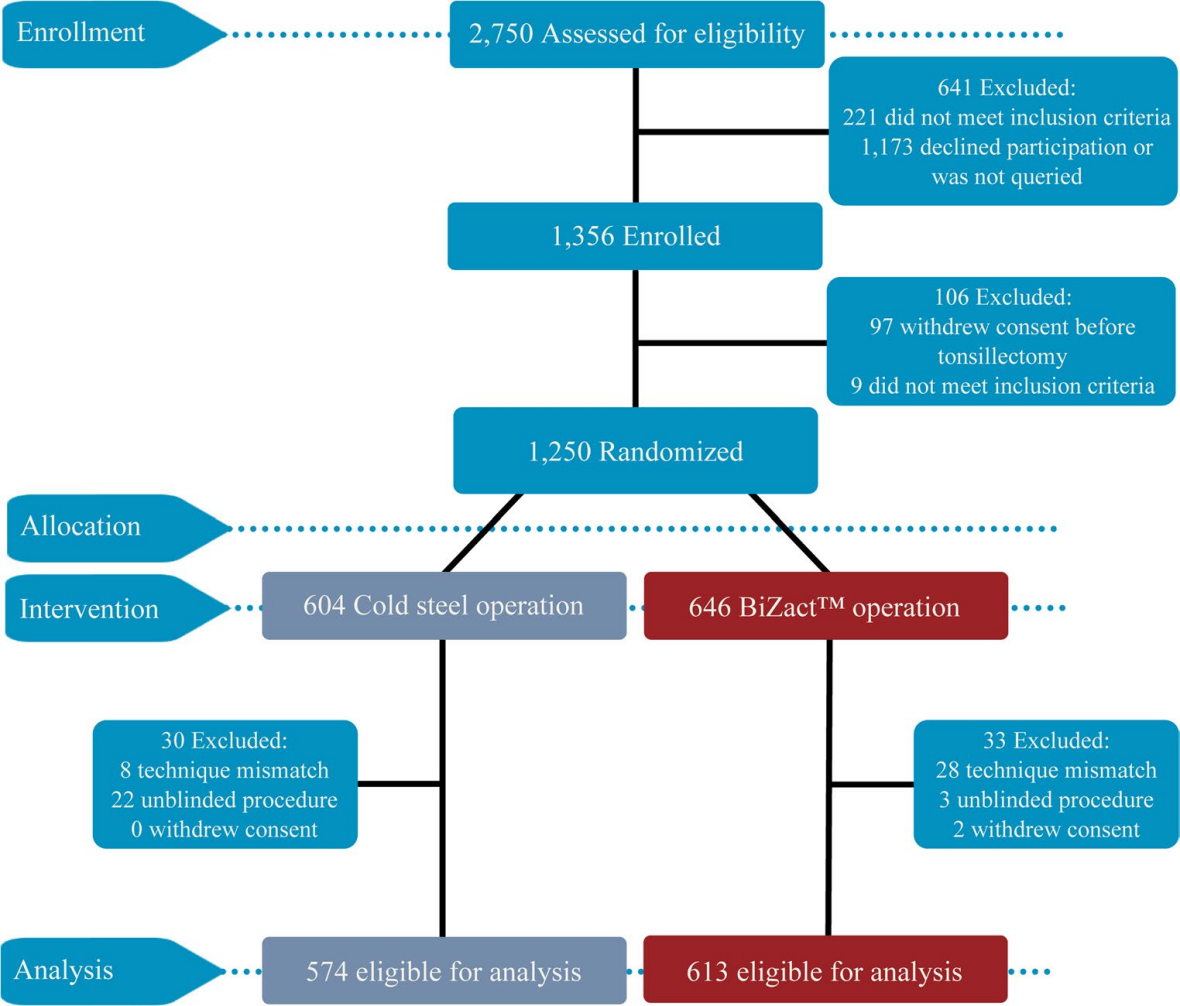
CONSORT flow diagram of the study cohort

Between June 1, 2022, and May 31, 2024, 2,750 patients were assessed for eligibility. A total of 1,356 were enrolled, of whom 1,250 were randomized: 645 to BiZact and 605 to cold steel tonsillectomy. After exclusions due to withdrawal of consent (n = 97) and failure to meet inclusion criteria (n = 9), 574 patients in the cold steel group and 613 in the BiZact™ group were included in the final analysis (Fig. 1).

Baseline characteristics were comparable across groups (Table 1). The cohort was predominantly female and young, with low rates of smoking and comorbidity. The most common surgical indications were recurrent tonsillitis and tonsillar hypertrophy. Most procedures were performed as outpatient surgeries. Resident surgeons conducted 63% of cold steel and 64% of BiZact tonsillectomies.

**Table 1:** Demographic characteristics of patients and non-participants

	Cold Steel group	BiZact group	Enrolled and later excluded patients
Total	574	613	106
Age in years (median, IQR)	21 (17–31)	22 (18–32)	23 (18–31)
Female sex (No. (%))	356 (62)	401 (65)	77 (73)
Smokers (No. (%))	71 (12)	69 (11)	14 (13)
BMI (median, IQR)	24 (20–29)	24 (21–30)	24 (22–28)
Physical comorbidity (No. (%))			
None	374 (65)	410 (67)	66 (62)
Asthma	35 (6)	33 (5)	2 (2)
Obesity (BMI ≥ 30)	123 (22)	145 (25)	19 (18)
Immunodeficiency	3 (0.5)	0 (0)	0 (0)
Diabetes mellitus type I or II	7 (1)	10 (2)	1 (1)
Neuromuscular disease	1 (0.2)	4 (0.7)	1 (1)
Congenital malformations or chromosomal abnormalities	0 (0)	0 (0)	1 (1)
Other ^a^	58 (10)	49 (8)	14 (13)
Indications for tonsillectomy (up to 2 per patient) (No. (%))			
Tonsillar hypertrophy	179 (31)	180 (29)	23 (22)
Recurrent tonsillitis	327 (57)	324 (53)	63 (59)
Peritonsillar abscess	27 (5)	27 (4)	1 (1)
Chronic tonsillitis	117 (20)	138 (23)	24 (23)
Systemic complications to tonsillitis	0 (0)	1 (0.2)	0 (0)
PFAPA ^b^	0 (0)	0 (0)	0 (0)
Tonsillar plugs	155 (27)	178 (29)	32 (30)
Residual tonsil tissue	1 (0.2)	0 (0)	1 (1)
Obstructive sleep apnea	64 (11)	71 (12)	5 (5)
Previous peritonsillar abscess	12 (2)	11 (2)	3 (3)

^a^ : Mainly consisting of psychiatric disorders, chronic pain, and hypertension

^b^ : PFAPA: periodic fever, aphthous stomatitis, pharyngitis, cervical adenitis

### Operative characteristics

BiZact tonsillectomy was associated with significantly shorter operative time (median 20 minutes (IQR 15–30)) compared to cold steel (31 minutes (IQR 24–40); p < 0.001) and significantly lower intraoperative blood loss (5 mL (IQR 1–20) versus 65 mL (IQR 32–116); p < 0.001) (Table 2). In 131 BiZact and 28 cold steel procedures, negative values from saline irrigation were adjusted to 0.1 ml.

**Table 2:** Operative parameters. Numbers are no. (%) unless otherwise specified

	Cold Steel group	BiZact group	*p*-value
Total	574	613	–
Outpatient procedures	406 (71)	432 (71)	–
Inpatient procedures	168 (29)	181 (30)	–
Type of procedure			
Elective tonsillectomy	462 (81)	499 (81)	–
Elective adenotonsillectomy	94 (16)	92 (15)	–
Acute peritonsillar abscess	18 (3)	22 (4)	–
Hemostasis technique			
No hemostasis intervention	3 (0.5)	130 (21)	< 0.001
Compression	515 (90)	411 (67)	< 0.001
Adrenaline infiltration	57 (10)	57 (9)	0.80
Unipolar diathermy	10 (2)	2 (0.3)	0.03
Bipolar diathermy	516 (90)	212 (34)	< 0.001
Ligature	41 (7)	5 (0.8)	< 0.001
Suture	19 (3)	4 (0.7)	0.002
Surgeon-grade			
ENT attending surgeon	213 (37)	222 (36)	1.00
ENT resident	361 (63)	391 (64)	–
Operation time (min) (median, IQR)	31 (24–40)	20 (15–30)	< 0.001
Intraoperative bleeding (ml) (median, IQR)	65 (32–116)	5(1–20)	< 0.001

An exploratory analysis of blood loss severity, categorized by estimated blood volume, revealed that all cases were Class I hemorrhages (<15% estimated volume) except for one Class II case in a cold steel patient under 12 years of age [24]. Due to the uniformity of Class I classification, no between-group analysis was conducted (Table S2).

### Post-tonsillectomy hemorrhage

A total of 225 PTH events were recorded and classified by severity (Table 3). Return to theatre under general anesthesia occurred in 5% of BiZact patients and 10% of cold steel patients (p = 0.004). Bleeding managed in local anesthesia occurred in 7% in both groups (p = 0.52). Bleeding with spontaneous hemostasis occurred in 3% of BiZact and 5% of cold steel patients (p = 0.15).

**Table 3:** Post-tonsillar hemorrhage (PTH), infection, and admission due to pain. Numbers are no. or no. (%)

	Cold Steel group	BiZact group	*p*-value	
Total PTH events	134	91	< 0.001	
Patients with PTH events ^a^	97 (17)	74 (12)	0.02	
PTH events with spontaneous hemostasis	32	20	0.06	
Patients with PTH with spontaneous hemostasis ^a^	27 (5)	18 (3)	0.15	
PTH events requiring intervention	102	71	0.006	
Patients with PTH requiring intervention	79 (14)	56 (9)	0.02	
All hospitals ^a^	79/575 (14)	56/612 (9)	0.02	
Gødstrup Hospital ^b^	36/216 (17)	31/213 (15)	0.64	
Aalborg University Hospital ^b^	17/76 (22)	9/87 (10)	0.06	
Regional Hospital of Southern Jutland, Sønderborg ^b^	12/106 (11)	9/130 (7)	0.34	
Lillebælt Regional Hospital, Vejle ^b^	10/81 (12)	5/90 (6)	0.19	
University Hospital of Southern Denmark, Esbjerg ^b^	4/96 (4)	2/92 (2)	0.68	
Type of PTH				
Primary	31	8	0.009	
Secondary	103	82	0.02	
Surgeon grade ^a^				
ENT attending surgeon	34 (16)	28 (13)	0.18	
ENT resident	61 (17)	46 (12)	–	
PTH hemostasis technique				
Local anesthesia	44	40	0.52	
General anesthesia	58	31	0.004	
Suture used	4	2	0.44	
Tranexamic acid	21	18	0.62	
Transfusion of blood elements	0	0	1.00	
Infection requiring antibiotics ^a^	12 (2)	11 (2)	0.88	
Infection requiring admission ^a^	1 (0.2)	4 (0.7)	0.38	
Readmission due to pain ^a^	4 (0.7)	7 (1)	0.55	

^a^ : Percentage is relative to total number of operated patients

^b^ : Percentage is relative to total number of operated patients at the respective hospital

**Fig. 2:**
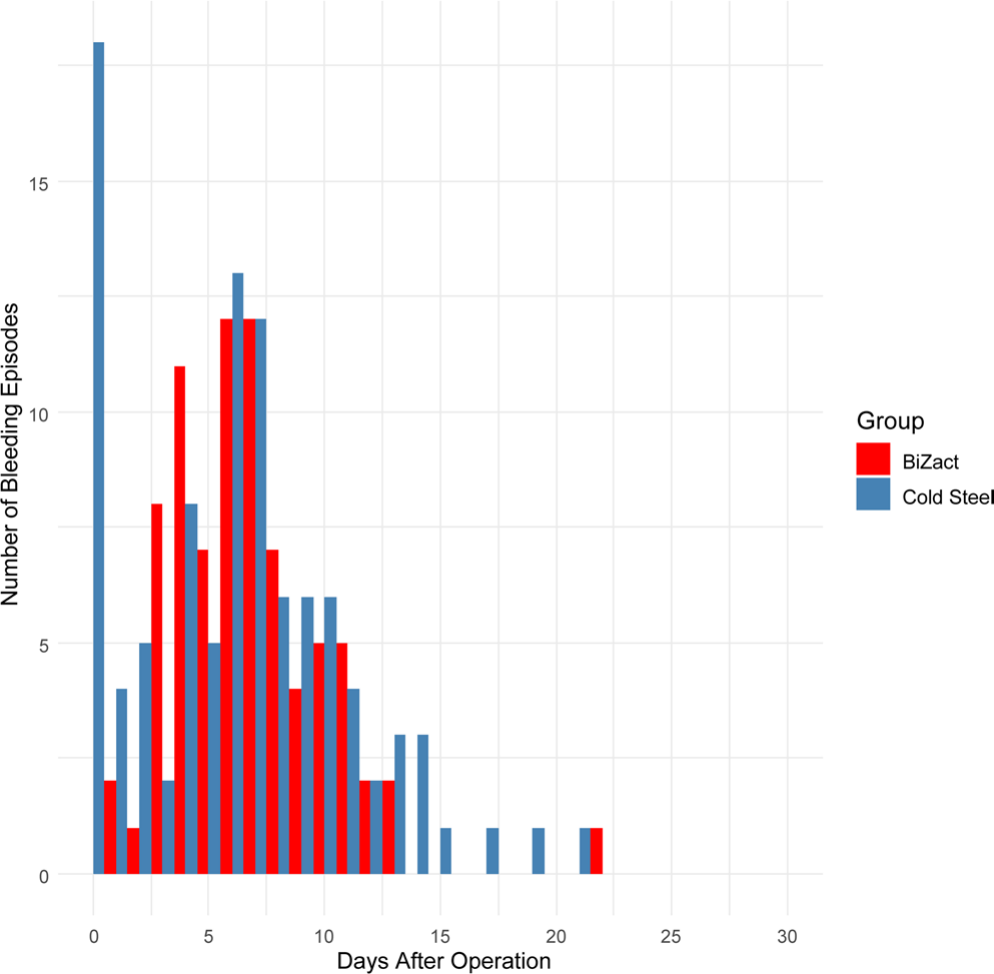
Histogram of bleeding cases by days after operation

When all PTH events were combined, the overall PTH rate was 12% in the BiZact group and 17% in the cold steel group (p = 0.02). The difference was driven primarily by a significantly lower rate of primary PTH episodes in the BiZact group (8 versus 31; p = 0.009). Secondary PTH episodes were also fewer in the BiZact™ group (82 versus 103; p = 0.02), though they remained the most common type in both arms. A histogram of bleeding by postoperative day showed a peak between days 5 and 7 (Fig. 2).

Adjusted Poisson regression with robust standard errors showed that BiZact was associated with a significantly lower risk of overall PTH (adjusted RR: 0.70; 95% CI: 0.53–0.92). The risk of primary PTH was also significantly lower with BiZact (adjusted RR: 0.26; 95% CI: 0.12–0.55), whereas the risk of secondary PTH did not differ between groups (adjusted RR: 1.04; 95% CI: 0.74–1.46) (Table 4).

**Table 4:** Risk of post-tonsillar hemorrhage (PTH), infection, and admission due to pain

	Cold Steel group			BiZact group		
	Rate^a^	Unadjusted RR (95% CI)	Adjusted RR^b^ (95% CI)	Rate^a^	Unadjusted RR (95% CI)	Adjusted RR^b^ (95% CI)
Overall PTH	0.17	1.0 (ref)	1.0 (ref)	0.12	0.72 (0.53–0.97)	0.70 (0.53–0.92)
Primary PTH	0.05	1.0 (ref)	1.0 (ref)	0.01	0.24 (0.11–0.53)	0.26 (0.12–0.55)
Secondary PTH	0.12	1.0 (ref)	1.0 (ref)	0.11	0.94 (0.67–1.32)	1.04 (0.74–1.46)
PTH risk according to procedure type^c^						
Elective tonsillectomy	0.16	1.0 (ref)	1.0 (ref)	0.13	0.80 (0.57–1.11)	0.79 (0.59–1.06)
Elective adenotonsillectomy	0.12	1.0 (ref)	1.0 (ref)	0.08	0.64 (0.25–1.60)	0.57 (0.24–1.40)
Acute peritonsillar abscess	0.15	1.0 (ref)	1.0 (ref)	0.18	1.18 (0.30–4.73)	0.98 (0.24–3.92)
Hemostasis technique						
Cold	0.10	1.0 (ref)	1.0 (ref)	0.12	1.47 (0.58–3.73)	1.56 (0.61–3.98)
Hot	0.17	1.0 (ref)	1.0 (ref)	0.13	0.80 (0.52–1.23)	0.60 (0.41–0.89)
Surgeon-grade						
ENT attending surgeon	0.17	1.0 (ref)	1.0 (ref)	0.13	0.89 (0.56–1.41)	0.75 (0.48–1.18)
ENT resident	0.17	1.0 (ref)	1.0 (ref)	0.12	0.74 (0.52–1.06)	0.70 (0.50–0.98)
Risk of infection	0.02	1.0 (ref)	1.0 (ref)	0.02	0.86 (0.38–1.95)	0.32 (0.67–1.57)
Risk of admission due to pain	0.007	1.0 (ref)	1.0 (ref)	0.01	1.64 (0.48–5.17)	0.79 (0.29–2.13)

^a^ : Rates are in relation to total number of operated patients

^b^ : Adjusted for male sex, age, smoking, any registered comorbidity, hospital, and tonsillectomy indications

^c^ : Not adjusted for tonsillectomy indications

### Subgroup and center-level analyses

No difference in overall PTH was observed between procedures performed by residents and attendings (p = 0.18). However, residents using BiZact had a significantly lower adjusted RR of PTH (0.70; 95% CI: 0.50–0.98), whereas no difference was seen among attendings (adjusted RR: 0.89; 95% CI: 0.56–1.41).

Center-level analysis showed that PTH rates were lower at Esbjerg (adjusted RR: 0.26; 95% CI: 0.15–0.47) and Sønderborg (adjusted RR: 0.65; 95% CI: 0.44–0.96) compared to the remaining centers. Excluding these two centers, BiZact remained associated with a significantly reduced PTH risk (adjusted RR: 0.72; 95% CI: 0.53–0.97) (Table S3).

PTH risk did not differ according to procedure type (elective tonsillectomy, adenotonsillectomy, or acute PTA drainage). When stratified by hemostasis technique, BiZact was associated with lower PTH risk only among cases using hot techniques (adjusted RR: 0.60; 95% CI: 0.41–0.89). No difference was observed when cold techniques were used.

### Secondary outcomes

Postoperative infection requiring antibiotic therapy occurred in 2% of both groups (p = 0.88), with no significant difference in infection-related admissions. Readmission due to pain occurred in 0.7% of cold steel and 1% of BiZact™ patients (p = 0.55). No transfusions or deaths were reported.

## Discussion

This multicenter randomized controlled trial found that BiZact tonsillectomy was associated with a significantly lower risk of PTH compared to cold steel dissection. The rate of return to theatre, used as a marker of severe PTH, was halved in the BiZact group (5% versus 10%). The overall PTH rate, including all events regardless of severity, was also significantly lower with BiZact (12% versus 17%). No differences were observed in postoperative infection rates or readmissions due to pain.

To our knowledge, this is the largest RCT on tonsillectomy to date. While several studies have compared cold and hot tonsillectomy techniques, few randomized trials have specifically examined BiZact. A recent meta-analysis found no significant reduction in PTH with BiZact compared to other methods [16]. This discrepancy may reflect the larger sample size of the current study. Notably, the trials by Besser et al. [18] and Manheim et al. [19] were limited by small cohorts and were powered for operative time and analgesic use rather than hemorrhage risk.

Our findings also differ from those of Russo et al. [7] who reported a PTH rate of 9% for the cold steel technique. However, many prior studies did not systematically capture bleeding events managed outside the operating theatre. In our study, all events were confirmed by ENT surgeons and classified by severity, providing a more complete bleeding profile. The comparatively high overall PTH rates of 12% and 17% may in part reflect the detailed documentation of all bleeding events, including those that were self-limiting or managed conservatively. This likely facilitated more complete detection of minor bleeds, which may partly account for the high PTH rates.

Primary PTH is often linked to technical errors or inadequate hemostasis [25, 26]. The lower rate of primary PTH in the BiZact group may be explained by the device’s ability to deliver focused thermal energy with minimal spread. This may improve hemostasis, particularly in less experienced hands. This hypothesis is supported by our finding that BiZact tonsillectomy performed by residents had significantly lower PTH rates than cold steel tonsillectomy, whereas no difference was observed among attendings. Interestingly, while crude PTH rates did not differ between residents and attendings, despite previous findings of lower bleeding rates with surgical experience [11], the stratified analysis suggests that BiZact may help reduce the influence of individual surgical skill on bleeding outcomes. Together, these findings contrast with earlier literature on surgical experience and reinforce the importance of instrument choice in influencing bleeding outcomes, including in the context of surgical training.

These results support BiZact as a safe and effective alternative ton cold steel tonsillectomy, particularly in high-volume or training settings where bleeding risk is a concern. The device was also associated with reduced operative time and lower intraoperative blood loss, which may improve surgical efficiency. Although the overall rate of secondary PTH did not differ between groups, significantly fewer cases required return to theatre under general anesthesia in the BiZact group. This suggests a shift toward less severe bleeding cases, with a higher proportion of secondary PTH episodes resolving spontaneously or being managed in local anesthesia. The findings of this trial may suggest that BiZact could be adopted as an alternative to cold steel, taking into account operative efficiency, bleeding risk, sustainability, and device cost.

When stratified by hemostasis technique, BiZact was associated with a significantly lower PTH risk only in procedures using hot hemostasis. No difference was observed between BiZact or cold steel tonsillectomy when cold hemostasis techniques were used. These findings align with prior registry-based analyses, including Söderman et al. [6] which have demonstrated increased bleeding risk when combining cold dissection with hot hemostasis. In our study, bipolar diathermy was used in 90% of cold steel and 34% of BiZact procedures. The differential use of hemostasis modalities may partially explain PTH differences and should be further investigated within Danish surgical practice.

Our study also emphasizes the importance of comprehensive postoperative surveillance. Many previous cohort studies have not reported self-limiting or outpatient-managed events, possibly resulting in an underestimation of the true PTH incidence.

### Strengths and limitations

The main strengths of this study include its randomized design, multicenter participation, and large sample size. Blinding of participants and care providers minimized risk of bias, and systematic record review ensured comprehensive outcome capture. The age and sex distribution matched a prior Danish national study, confirming cohort representativeness [1].

Limitations include the loss to follow-up of 30 cold steel and 33 BiZact patients, though this was evenly distributed. Technique mismatches mainly occurred in the cold steel group, while unblinded procedures were more common in the BiZact group due to inclusion oversights. Surgeon-level clustering could not be modeled due to data constraints, although center-level variance was low. Institutional variability in the choice of anesthesia for PTH may have affected the classification of event severity. Finally, as the study was conducted within the Danish healthcare system, generalizability to other countries with different surgical protocols may be limited.

## Conclusions

In this multicenter RCT, BiZact tonsillectomy was associated with a 50% lower risk of return to theatre for PTH and 30% reduced overall PTH risk compared to cold steel dissection. These results support BiZact as a safe and efficient alternative, particularly in settings prioritizing operative time, hemostatic control, and safety in training. Future trials in non-Danish healthcare systems are warranted to assess the external validity of these findings and evaluate how differences in hemostasis techniques may influence outcomes in other healthcare systems.

## Supplementary Information

Below is the link to the electronic supplementary material.

ESM 1 (DOCX 43.5 KB)

ESM 2 (DOCX 644 KB)

ESM 3 (DOCX 23.2 KB)

## Data Availability

The data upon which this study is based will be available from the corresponding author upon reasonable request.
