# Supplementary material for: BiZact versus cold steel for post-tonsillectomy hemorrhage: a multicenter randomized trial
Source: Eur Arch Otorhinolaryngol. 2025 Oct 13;282(12):6449–57. doi: 10.1007/s00405-025-09703-3 (PMC12680691; doi:10.1007/s00405-025-09703-3)
Supplement: Supplementary file 2 — (DOCX 644 KB) [file 405_2025_9703_MOESM2_ESM.docx]

**Table 1:** Overview of measurements, timing, and definitions.

**Table 2:** Overview of data entered in the online register by both physician and patients.

**Figure 1:** Participating ear, nose and throat departments in Jutland are: Aalborg University Hospital and Thisted Hospital. Gødstrup Hospital. Esbjerg Hospital. Lillebælt Hospital, Vejle. Sønderjyllands Hospital, Sønderborg.

**Figure 2:** Eligible patients who have signed a written consent are included in the study. Patient information is entered into the tonsil database by the surgeon. The computer randomly assigns patients to two study arms via block randomization. The control group undergoes standard cold steel tonsillectomy. The investigational group receives tonsillectomy with impedance dependent tissue sealer device. The per-operative procedure is equal for both groups as well as postoperative recommendations. The patients or their caregivers answer daily questionnaires on their mobile device regarding adherence to postoperative recommendations; pain scores on a Likert scale for adults/Wong-Baker scale for children; activity level; food consumption; unscheduled contacts to the health care system; return to school, work or childcare; satisfaction with the procedure; health related quality of life on the EQ5D-SL questionnaire; and quality of taste and smell. If post-tonsillectomy hemorrhage, infection, rehospitalization for rehydration occurs the doctor will input a detailed account in the database.

**Randomized controlled trial on Cold Steel versus Impedance Dependent Tissue Sealer tonsillectomy comparing postoperative morbidity**

Authors: Schiøtt Nissen, Line^1^; Bertelsen, Jannik^1^; Lyhne, Nina Munk^3^; Holm Nielsen, Susanne^4^; Hahn, Pernille^5^; Holm, Jesper^6^; Ovesen, Therese^1,2^.

Affiliations: 1 University Clinic for Balance, Flavour and Sleep, ENT department Gødstrup, Hospitalsparken 15, DK-7400 Herning, 2 Department of Clinical Medicine, Aarhus University, Palle Juul-Jensens Boulevard 82, DK-8200 Aarhus N, 3 Ear, nose and throat department, Aalborg University Hospital, Hobrovej 18-22, DK-9000 Aalborg; 4 Ear, nose and throat department, Sygehus Sønderjylland, Sydvang 1, DK-6400 Sønderborg, 5 Ear, nose and throat department, Sygehus Lillebælt, Beriderbakken 4, DK-7100 Vejle, 6 Ear, nose and throat department, Sydvestjysk Sygehus, Finsensgade 35 DK-6700 Esbjerg.

**Introduction**

Tonsil surgery, with or without adenoidectomy, performed on both children and adults is one of the most frequent surgical procedures in the ear, nose and throat (ENT) specialty worldwide, and the number of tonsil operations has increased over recent decades^1–5^. In Denmark, approximately 7.000 tonsil operations are performed annually^6^ based on benign indications such as recurrent episodes of acute tonsillitis and upper airway obstruction due to adeno-tonsillar hypertrophy^7^.

Although, commonly performed, tonsil surgery is associated with significant morbidity^3^, such as postoperative pain and more severely post tonsillectomy hemorrhage (PTH)^7–12^. Postoperative pain lasts for approximately two weeks, and may in severe cases result in delayed discharge, a visit at the emergency department or readmission for pain control, hydration, and treatment of possible underlying infection^3^. The incidences of PTH vary between 0.3 and more than 10%^1,11,13,14^ and is a major and potential life-threatening complication of tonsil surgery^3,8^.

Because the evidence base of tonsil surgeries in Denmark is low, it is recommended in the Danish National Clinical Guideline from 2019^15^ , to secure the monitoring of tonsil surgeries via a uniform registration of procedures and diagnoses in Denmark. Further recommendations^15^ are that tonsil surgeries are monitored via a database, that can register the surgical method, the extent of the surgery (tonsillectomy vs. tonsillotomy), and length of postoperative hospital stay which is not properly described in Danish patients^15^. The criteria for offering patients tonsil surgery vary in Denmark, and the diagnostic criteria are not well-defined for especially recurrent acute tonsillitis and chronic tonsillitis^15^. There is also believed to be a variation in the applied surgical techniques especially on which instruments are used for the removal. Such data can be obtained from properly designed clinical databases^16^.

The Danish Tonsil database is a population based clinical database established in 2017 in the Central Denmark Region. It is a copy of the concept underlying The National Tonsil Surgery Register in Sweden (NTSRS), which was founded in 1997 covering more than 80% of all tonsil surgeries in Sweden. Several retrospective studies based on data from the register have been published^8,17^.

From 2021, ENT departments of the Region of Southern Denmark, and the North Denmark Region have entered the Danish tonsil database collaboration. The database contains pre-, per-, and postoperative information about tonsil surgery, including surgical method and instruments.

Although cold steel tonsillectomy is considered the gold standard surgical technique, several hot techniques including bipolar techniques, diathermy, and coblation have been developed for tonsillectomy^2^. Hitherto, these hot instruments have not proven superior to the gold standard in terms of reducing PTH, and it is recommended, that these hot techniques should be used with caution^2,3,5,8,18,19^. In cold steel tonsillectomy the peritonsillar space is dissected with metal instruments, and bleeding is typically controlled by ligation or electrocautery.

Recently, a new impedance-dependent tissue sealer (IDTS) device (BiZact^TM^) has been approved for all benign indications for tonsillectomy^20^. Preliminary results are promising in terms of a PTH rate of 4.5%, a shortened operative time with a median time of 5.1 minutes (range 1.5-26.5 minutes), and a reduced intraoperative blood loss to 1-10 milliliters^20^. With the IDTS the peritonsillar space is dissected and sealed in one step using adjustable energy from a ValleyLab FT10 Energy Platform (Medtronic) measuring the impedance of the tissue grasped with the instrument securing hemostasis before the surgeon cuts the tissue.

Ideally, if a gold standard technique is to be replaced by a new technique, it is recommended that the decision should be based on scientific studies performed as randomized designs.

**Primary objective**

The aim of the study is to evaluate whether tonsillectomy performed with an IDTS is non-inferior compared to tonsillectomy performed with cold steel instruments in terms of PTH and postoperative pain.

**Method**

*Design*

The project is designed as a Danish randomized controlled multi-center trial of tonsillectomy by conventional cold steel surgical technique versus IDTS. The study is blinded for patients until six months after surgery and the study is assessor blinded.

Randomization is computerized via the tonsil database and utilizes block randomization for each department in a random table of 2000 with equal numbers of group assignments. The randomization is only apparent to the surgeon on the day of the surgery and the day before.

*Course*

All elective and acute patients referred to tonsillectomy, with or without adenoidectomy, on benign indications will be assessed for eligibility at the participating ENT departments. Given a signed declaration of consent patients will be enrolled and randomized in the study to either the standard cold steel tonsillectomy or to IDTS tonsillectomy.

After surgery all patients will be brought to the recovery room for 1-4 hours or hospitalized for a day. Before hospital discharge a surgeon will check for postoperative hemorrhaging and the patient and caregivers will receive oral and written recommendations for the postoperative period. Patients or caregivers are requested to follow the recommendations, and they are instructed to contact the ENT department or to call an ambulance, if postoperative hemorrhage occurs.

There will be no planned postoperative visits at the hospital. During the first six postoperative months included patients/caregivers are asked to complete electronic questionnaires online on their mobile device: daily, for the first two postoperative weeks, and again one month and six months postoperatively.

**Outcome Assessment**

Primary endpoints:

- Incidence of PTH defined as hemorrhage requiring hemostasis with bipolar electric coagulation, ligature, compression and/or medical treatment with anti-fibrinolytic within the first 24 hours after surgery (early PTH) until 30 days after surgery (late PTH).
- Intraoperative blood loss assessed by a standardized protocol utilizing the gravimetric method. The nurse suctions up 100 ml of NaCl weighed on the scale for precision (Kern & Sohn, PCB 6000-1). The suction canister is also weighed on the scale, as well as the dry gauze. After the surgery the canister of blood including the NaCl is weighed and the dry weights of the canister and gauze including the NaCl are subtracted, this leaves the total blood loss of the operation. It is assumed that 1 gram blood = 1 ml blood.
- Operative time in minutes from the Boyle-Davis gag is placed until it is removed. The operating nurse is responsible for measurements of time.
- Postoperative pain is assessed on an 11-point numeric rating scale ranging from 0 “no pain” to 10 “worst possible pain”. For children, the Wong-Baker Faces rating scale will be used.

Secondary endpoints:

- Patient reported: Unscheduled contacts to the health care system, patient satisfaction, health related quality of life, and patient reported quality of smell and taste via a validated TASTE questionnaire, followed by further investigation should the patient report altered taste at six months.

Variables:

- Patient reported outcome measures (PROMs) of postoperative recovery (return to normal diet, physical activity, and return to work, school, or childcare), compliance to postoperative recommendations.

**Inclusion criteria:** First time tonsillectomy due to; Tonsillar hypertrophy, recurrent tonsillitis (including previous peritonsillar abscess), chronic tonsillitis, systemic complications to tonsillitis, other (mononucleosis), PFAPA (periodic fever, aphthous stomatitis, pharyngitis, cervical adenitis), tonsillar plugs, peritonsillar abscess and signed written consent.

**Exclusion criteria:** Diseases in the hematopoietic system, antithrombotic or anticoagulant drugs in the recovery period, suspicion of or known malignancy, patients, or parents unable to read or speak Danish, patients, or parents unable to use online application for self-evaluation, patients or parents unable to give informed consent, body weight under 16 kilograms.

**Power Considerations:** The sample size calculation is based on data from the Danish Tonsil Database. In a twelve months period, the frequency of PTH causing reoperation with hemostasis was 7.9%. With an expected non-inferiority treatment difference between the IDTS and the cold steel instrument, a significance level of 0.05, a power of 80%, and a non-inferiority limit of 4%, the required sample is 1126 patients. Assuming a drop out frequency of 10% into account, a total of 1250 patients are required. With a non-inferiority limit set to 4%, the present study will be able to detect a clinically relevant change in the frequency of PTH causing reoperation with hemostasis.

**Statistical analysis**

Endpoints will be evaluated as differences between treatment groups. Categorical data are analyzed by chi2 or Fisher’s exact test. Depending on normal distribution, continuous data is analyzed by either un-paired Student’s *t-test* or Mann-Whitney rank sum test. An Intention-to-treat principle will be used including all randomized participants in the analysis. Stratification will be performed, and confounding factors (sex, age, surgical indication, BMI, smoking, comorbidity) analyzed in a multiple logistic regression model. Repeated measurement data will be analyzed in a mixed effect model for repeated measurements (ANOVA) with group and time as systematic factors and patients as random effect. The model takes random variations over time into account. Post-hoc tests will be based on the Kenward-Roger approximation to test all included patients despite missing data. The significance level is set at 0.05.

**Ethical Considerations and project feasibility**

The study is reported and approved by the Danish Data Protection Agency, case number 1-16-02-152-22 and the Medical Research Ethics Committee, case number 2202151.

The study will be conducted according to Helsinki declaration. Participation in the study is voluntary. The participants will receive oral and written information and be guaranteed confidentiality. Written consent will be received from all patients. The study is registered on clinicaltrials.gov with the identification number NCT05270109.

The collaborating departments have the necessary professional competence, capacity and equipment, and each site has a co-investigator. All ENT departments have signed cooperation agreements, reference number 1-10-81-95-21 and data processor agreements, case number of the main agreement 1-52-81-216-21.

**Safety:** It is not expected that the intervention under investigation will cause risks or side effects other than the well-known related to tonsil surgery. All unexpected peri- and postoperative complications and adverse events will be registered. Furthermore, an interim analysis will be conducted after inclusion of 650 patients. Should the results of the interim analysis reveal that one of the interventions is associated with significant adverse events, the study will be terminated.

**Data sharing:** The data resulting from this study will be available from the corresponding author upon reasonable request.

**Discussion**

This protocol describes a Danish randomized controlled multicenter trial with the primary aim to evaluate whether IDTS tonsillectomy is non-inferior compared to cold steel tonsillectomy with regards to PTH and pain.

The study has four key strengths. The study is a randomized controlled trial with block randomization securing that patients are consecutively enrolled in the surgical treatment groups, a vital step to avoid selection bias. A further key strength is the multicenter setup considered the ideal way to generate data ready for generalization and enabling the enrollment of the large number of participants required for the protocol. In this study the multicenter setup entails cooperation of three regions including five treatment centers, hereunder as well secondary and tertiary treatment centers. The setup ensures a wide population-based inclusion in the project. Furthermore, the project will be both assessor blinded and blinded for the patients to prevent observer and confirmation bias.

There are also limitations to this study. The most significant limitation of the study is the lack of definition of PTH worldwide. The literature thoroughly describes primary and secondary bleeding occurring before and after 24 hours, respectively, but without a clear definition of severity. In this study we will define severity by interventions needed for PTH to accommodate this.

A potential limitation for the study is also one of the strengths - the multicenter setup inhabits a risk of one center not enrolling to equal measure of other centers, thereby entailing a selection bias. To remedy the selection bias of the center the dataset will potentially have to be excluded, which will weaken the results. The comprehensive logistics of multicenter studies sometimes result in unexpected heterogeneity in clinical practices between centers, which may induce confounders affecting the interpretation of the results.

In our study we have daily patient reported outcomes on pain management, which potentially will alter the patients’ compliance, also called the Hawthorne effect.

To our knowledge, the present study is the largest randomized controlled trial in ENT surgery in the Nordic countries to test the hypothesis whether IDTS tonsillectomy is non-inferior to cold steel tonsillectomy with regards to PTH, surgery time and postoperative pain.

The study will contribute the current literature with knowledge regarding the clinical course after standard cold steel tonsillectomy and provide evidence regarding potential benefits of the IDTS technique compared to cold steel.

References

1. Mueller J, Boeger D, Buentzel J, et al. Population-based analysis of tonsil surgery and postoperative hemorrhage. *Eur Arch Otorhinolaryngol*. 2015;272(12):3769-3777. doi:10.1007/s00405-014-3431-6

2. Blanchford H, Lowe, D. Cold versus Hot Tonsillectomy: State of the Art and Recommendations. *ORL*. 2013;75(3):136-141. doi:10.1159/000342315

3. Pynnonen M, Brinkmeier JV, Thorne MC, Chong LY, Burton MJ. Coblation versus other surgical techniques for tonsillectomy. *Cochrane Database Syst Rev*. 2017;2017(8):1-100. doi:10.1002/14651858.CD004619.pub3

4. Erickson BK, Larson DR, St. Sauver JL, Meverden RA, Orvidas LJ. Changes in incidence and indications of tonsillectomy and adenotonsillectomy, 1970-2005. *Otolaryngol Head Neck Surg*. 2009;140(6):894-901. doi:10.1016/j.otohns.2009.01.044

5. Windfuhr JP, Chen YS. Do changing trends in tonsil surgery affect hemorrhage rates? A longitudinal study covering 1,452,637 procedures. *Eur Arch Otorhinolaryngol*. 2019;276(9):2585-2593. doi:10.1007/s00405-019-05532-3

6. Juul ML, Rasmussen ER, Rasmussen SHR, Sørensen CH, Howitz MF. A nationwide registry-based cohort study of incidence of tonsillectomy in Denmark, 1991-2012. *Clin Otolaryngol*. 2018;43(1):274-284. doi:10.1111/coa.12959

7. Baugh RF, Archer SM, Mitchell RB, et al. Clinical Practice Guideline: Tonsillectomy in Children. *Otolaryngol Neck Surg*. 2011;144(S1):S1-S30. doi:10.1177/0194599810389949

8. Söderman ACH, Odhagen E, Ericsson E, et al. Post-tonsillectomy haemorrhage rates are related to technique for dissection and for haemostasis. An analysis of 15734 patients in the National Tonsil Surgery Register in Sweden. *Clin Otolaryngol*. 2015;40(3):248-254. doi:10.1111/coa.12361

9. Tomkinson A, Harrison W, Owens D, Harris S, McClure V, Temple M. Risk factors for postoperative hemorrhage following tonsillectomy. *The Laryngoscope*. 2011;121(2):279-288. doi:10.1002/lary.21242

10. Tan GX, Tunkel DE. Control of Pain After Tonsillectomy in Children: A Review. *JAMA Otolaryngol Neck Surg*. 2017;143(9):937-942. doi:10.1001/jamaoto.2017.0845

11. Sarny S, Ossimitz G, Habermann W, Stammberger H. Hemorrhage following tonsil surgery: A multicenter prospective study. *The Laryngoscope*. 2011;121(12):2553-2560. doi:10.1002/lary.22347

12. Bhattacharyya N, Kepnes LJ. Revisits and postoperative hemorrhage after adult tonsillectomy. *The Laryngoscope*. 2014;124(7):1554-1556. doi:10.1002/lary.24541

13. Blakley BW. Post-tonsillectomy bleeding: How much is too much? *Otolaryngol Head Neck Surg*. 2009;140(3):288-290. doi:10.1016/j.otohns.2008.12.005

14. Windfuhr JP. Serious Complications following Tonsillectomy: How Frequent Are They Really? *ORL*. 2013;75(3):166-173. doi:10.1159/000342317

15. ISBN elektronisk udgave:978-87-7014-327-1. *Sundhedsstyrelsen. National Klinisk Retningslinje for Fjernelse Af Mandler (Tonsillektomi)- Sundhedsstyrrelsen. 2019.*; 2019.

16. Sørensen HT. Regional administrative health registries as a resource in clinical epidemiologyA study of options, strengths, limitations and data quality provided with examples of use. *Int J Risk Saf Med*. 1997;10 1:1-22.

17. Lundström F, Stalfors J, Østvoll E, Sunnergren O. Practice, complications and outcome in Swedish tonsil surgery 2009–2018. An observational longitudinal national cohort study. *Acta Otolaryngol (Stockh)*. 2020;140(7):589-596. doi:10.1080/00016489.2020.1746396

18. Gysin C, Dulguerov P. Hemorrhage after Tonsillectomy: Does the Surgical Technique Really Matter? *ORL*. 2013;75(3):123-132. doi:10.1159/000342314

19. Mowatt G, Cook JA, Fraser C, McKerrow WS, Burr JM. Systematic review of the safety of electrosurgery for tonsillectomy. *Clin Otolaryngol*. 2006;31(2):95-102. doi:10.1111/j.1749-4486.2006.01162.x

20. Krishnan G, Stepan L, Du C, et al. Tonsillectomy using the BiZact: A pilot study in 186 children and adults. *Clin Otolaryngol*. 2019;44(3):392-396. doi:10.1111/coa.13273
