# Supplementary material for: BiZact versus cold steel for post-tonsillectomy hemorrhage: a multicenter randomized trial
Source: Eur Arch Otorhinolaryngol. 2025 Oct 13;282(12):6449–57. doi: 10.1007/s00405-025-09703-3 (PMC12680691; doi:10.1007/s00405-025-09703-3)
Supplement: Supplementary file 3 — (DOCX 23.2 KB) [file 405_2025_9703_MOESM3_ESM.docx]

Amendments to trial protocol: Randomized controlled trial on Cold Steel versus Impedance Dependent Tissue Sealer tonsillectomy comparing postoperative morbidity

*Authors*The original protocol designated Schiøtt Nissen, Line, as the first author and primary investigator. However, these roles have since been replaced by Martin Mølhave.

*Statistical analysis*The original protocol specified stratification and analysis of confounding factors (sex, age, surgical indication, BMI, smoking, and comorbidities) using a multiple logistic regression model to calculate odds ratios. However, as the author group preferred relative risk as the risk measure, modified Poisson regression models were used instead to calculate relative risks.
